# Protopine triggers apoptosis via the intrinsic pathway and regulation of ROS/PI3K/Akt signalling pathway in liver carcinoma

**DOI:** 10.1186/s12935-021-02105-5

**Published:** 2021-07-27

**Authors:** Chunhui Nie, Bei Wang, Baoquan Wang, Ning Lv, Rui Yu, Enfan Zhang

**Affiliations:** 1 Hepatobiliary and Pancreatic Interventional Treatment Center, Division of Hepatobiliary and Pancreatic Surgery, The First Affiliated Hospital, School of Medicine, Zhejiang University, Hangzhou, Zhejiang China; 2 Key Laboratory of Combined Multi-Organ Transplantation, Ministry of Public Health, Hangzhou, Zhejiang China; 3 Department of Pharmacy, The First Affiliated Hospital, School of Medicine, Zhejiang University, Hangzhou, Zhejiang China; 4 Department of Biochemistry and Molecular Biology, School of Medicine, Ningbo University, No. 818, Fenghua Road, Ningbo, Zhejiang China; 5 Bone Marrow Transplantation Center, Department of Hematology, The First Affiliated Hospital, School of Medicine, Zhejiang University, No. 79, Qingchun Road, Hangzhou, Zhejiang China

**Keywords:** Protopine, Liver carcinoma, Apoptosis, ROS, PI3K/Akt

## Abstract

**Background:**

Protopine is an isoquinoline alkaloid that possesses various biological activities including the anti-tumour activity. However, the effects of protopine on liver carcinoma cells are still elusive. The aim of this study is to examine the effects of protopine on liver carcinoma cells both in vitro and in vivo.

**Methods:**

MTT assay was performed to measure the cell viability. Wound healing and transwell assays were conducted to assess the motility of cells. Cellular apoptosis and ROS levels were measured by the flow cytometry. Western blotting assay was used to measure the change of proteins. The cytotoxicity of protopine was also evaluated in xenograft mice.

**Results:**

Protopine inhibited viabilities and triggered apoptosis via the intrinsic pathway in a caspase-dependent manner in liver carcinoma cells. Furthermore, protopine also induced accumulation of intracellular ROS which further led to the inhibition of PI3K/Akt signalling pathway. Finally, in vivo study showed that protopine also repressed tumour growth in xenograft mice without noticeable toxicity.

**Conclusions:**

Protopine might be used as a potential therapeutic agent for the treatment of liver carcinoma.

## Background

Liver carcinoma is one of the most common tumours with extremely high morbidity and mortality worldwide [1]. Liver carcinoma, which has an increasing incidence every year, is the second leading cause of cancer-related death according to the statistics of World Health Organization [2]. Unfortunately, majority of the liver carcinoma cases were diagnosed at late stage and the surgery or transplantation is not suitable. Currently, chemotherapy is still the most widely used strategy for the treatment of advanced stage liver carcinoma. However, the application of chemotherapeutic agents is often hampered by unwanted severe toxicity effects on the normal cells. Hence, its necessary to identify novel anticancer agents/compounds to improve the prognosis of liver carcinoma.

In recent years, natural products got great attentions as an important source of novel agents for a wide range of diseased including the cancers. All the new drugs approved by the FDA from 1981 to 2019 (n = 1881) are natural products or natural products derivates [3]. Till now, numerous natural products showed promising activities against the liver carcinoma. For instance, DMAMCL, a natural plant-derived guaianolide sesquiterpene lactone, has been found inhibited the tumorigenesis of liver carcinoma both in vitro and in vivo [4].

Protopine is an isoquinoline alkaloid that can be found in a variety of plants such as Papaveraceae and Fumariaceae in the northeast part of Asia [5]. Similar to other natural alkaloids, protopine has been found to possess various biological activities such as anti-inflammation, anti-microbial, anti-angiogenic and anti-tumours [6]. Although protopine has been proved to exert anti-tumour activities against various cancers such as colon cancer, prostate cancer and pancreatic cancer [7–9]. However, the effects of protopine on the tumorigenesis of liver carcinoma has not been investigated yet. Considering that protopine also possesses the hepatoprotective activity in liver injury [10]. It would be interesting to study the effects and its underlying mechanisms of protopine on the liver carcinoma.

In the current study, we aimed to evaluate the effects of protopine on the tumorigenesis of liver carcinoma. It was found that protopine inhibited the viabilities and induced apoptosis via the mitochondrial pathway in liver carcinoma cells. Furthermore, it was also found that protopine inhibited the PI3K/Akt signalling pathway which relied on the generation of ROS. Finally, protopine could also inhibit the growth of liver carcinoma cells in vivo. Our data suggested that protopine might be developed into a chemotherapeutic agent for the treatment of liver carcinoma.

## Methods

### Cell culture and chemicals

The human liver carcinoma cell lines HepG2 and Huh7 were purchased from Shanghai Bank of Cell Culture, Shanghai Institute of Biological Sciences, Chinese Academy of Sciences (Shanghai, China). All cells were cultured in the RPMI1640 medium (Gibco, USA) supplemented with 10% FBS (fetal bovine serum, Gibco) and 1% penicillin/streptomycin (GIBCO) at 37 °C in a humidified incubator with 5% CO_2_. Caspase-3 inhibitor (Q-DEVD-OPh), caspase-8 inhibitor (Z-IETD-FMK), caspase-9 inhibitor (Z-LEHD-FMK) and pan-caspase inhibitor (z.VAD-FMK) were obtained from Abcam (USA). All other chemicals used were obtained from Sigma-Aldrich (USA).

### MTT assay

HepG2 and Huh-7 cells were seeded into 96-well plates at the density of 5000 cells/well. After treated with various doses of protopine under various conditions, cell viabilities were measured using the MTT assay (Sigma) as described previously [11].

### Wound healing assay

Cellular migration was measured using the wound healing assay. Cells were seeded into the 24-well plate at the density of 1 × 10^4^ cells/well. 24 h later, cells were cultured to 90% confluence, a scratch was created by a sterile tip on the monolayer cells and cells were treated with various doses of protopine. 24 h later, the width of wound was measured under an inverted microscope (Olympus, Japan) at × 100 magnification and normalized to initial distance at 0 h.

### Invasion assay

Transwell assay was performed to measure cellular invasion. Cells were suspended in serum-free medium containing various doses of protopine at the density of about 400 cells/μl and seeded into the upper chambers of transwell which was coated with the Matrigel (BD Biosciences, USA), 500 μl of full culture medium with 10% FBS was placed into the bottom chamber as the chemoattractant. After culture for 48 h, the cells remaining in the top chambers were removed and the cells in the bottom chambers were fixed with 4% paraformaldehyde and stained with 0.1% crystal violet. The number of invaded cells were counted under the inverted microscope (Olympus, Japan) at × 100 magnification.

### Cellular apoptosis assay

After being treated with various doses of protopine for 24 h, 1 × 10^5^ cells were collected and staining with 500 μl Annexin V binding buffer with 5 μl of Annexin V-FITC at room temperature for 0.5 h and avoid the light. Then cellular apoptosis was measured using the flow cytometer (BD Biosciences, USA).

### Relative caspase activities assay

The relative activities of caspase-3, caspase-8 and caspase-9 were measured by the caspase-3, caspase-8 and caspase-9 multiplex activity assay kit (Abcam, USA) according to the manufacturer’s guide. In brief, cells were treated with various doses of protopine for 24 h, and cellular lysates were collected. Equal amount of cellular lysates (10 μl) was added into 96-well plates and 80 μl of reaction buffer containing various caspase substrates. After incubated at 37 °C for 4 h, caspase activities were measured at the absorbance at 450 nm.

### ROS measurement

Levels of ROS were measured by staining cells with 2′7′-dichlorodihydrofluorescein diacetate (DCFH-DA; Sigma-Aldrich) according to the manufacturer’s guide. After treated with various doses of protopine for 24 h, then cells were incubated with 10 mM DCFH-DA for 0.5 h to avoid light, and fluorescence was detected by flow cytometry (BD Bioscience, USA).

### MDA, SOD, LDH and GPX assays

The levels of MDA, SOD, LDH and GPX were measured by the Lipid Peroxidation (MDA) Assay kit, SOD activity assay kit, LDH assay kit and Glutathione Peroxidase (GPX) Assay Kit, respectively according to the manufacturer’s guide. All kits were obtained from the Abcam.

### RNA sequencing analysis

Cells were treated with or without protopine for 24 h and total RNA was then extracted for RNA-seq in triplicate. RNA-seq was performed and analyzed by GeneChem Ltd (Shanghai, China). Various signalling pathways were investigated and the significant differentially expressed genes (P < 0.05) were classified into corresponding signalling pathways.

### Western blotting assay

Cells were harvested and lysed with RIPA buffer after transfection. The protein concentrations were assayed a Bradford protein assay kit (Beyotime). Equal amounts of protein (20 μg) were separated by 12% SDS-PAGE and transferred to PVDF membranes (Merck-Millipore Bioscience) at 100 V for 1 h. The blots were blocked with 5% skimmed milk at room temperature for 1 h, followed by incubation with different primary antibodies at 4 °C overnight. All antibodies were obtained from the Cellular Signalling Technologies (USA). Signals were visualized using an enhanced chemiluminescence (ECL; Pierce, USA).

### In vivo* study*

Male BALB/c mice at 6–8 weeks were chosen and randomly divided into four groups (n = 5/group). Cells were implanted (10^7^ cells/ml) subcutaneously into the right frontal axils of the mice. When the tumour volume reached about 100 mm^3^, the mice were treated with an intravenous injection of saline (control) or various doses of protopine. Tumor volume was measured once every 3 days and calculated by the formula: volume = (width^2^ × length)/2. At the end of the treatment, the tumours were resected and the weight of tumours were measured. Then the tumours protein was extracted using the One-Step animal tissue active protein extraction kit (Sangon Biotech, Shanghai, China) according to the manufacturer’s guide. All animal experiments followed ethical standards and all protocols were approved by the animal using committee of Zhejiang University.

### Statistical analysis

Data were analysed using SPSS 12.0 software (Chicago, IL, USA). All experiments were conducted at least three times. The results are expressed as the mean ± SD. Difference between groups was compared using One-way ANOVA followed Tukey’s post-hoc test. A value of p < 0.05 was considered significantly different.

## Results

### Protopine inhibited the viability, migration, invasion and EMT process of liver carcinoma cells

**Fig. 1:**
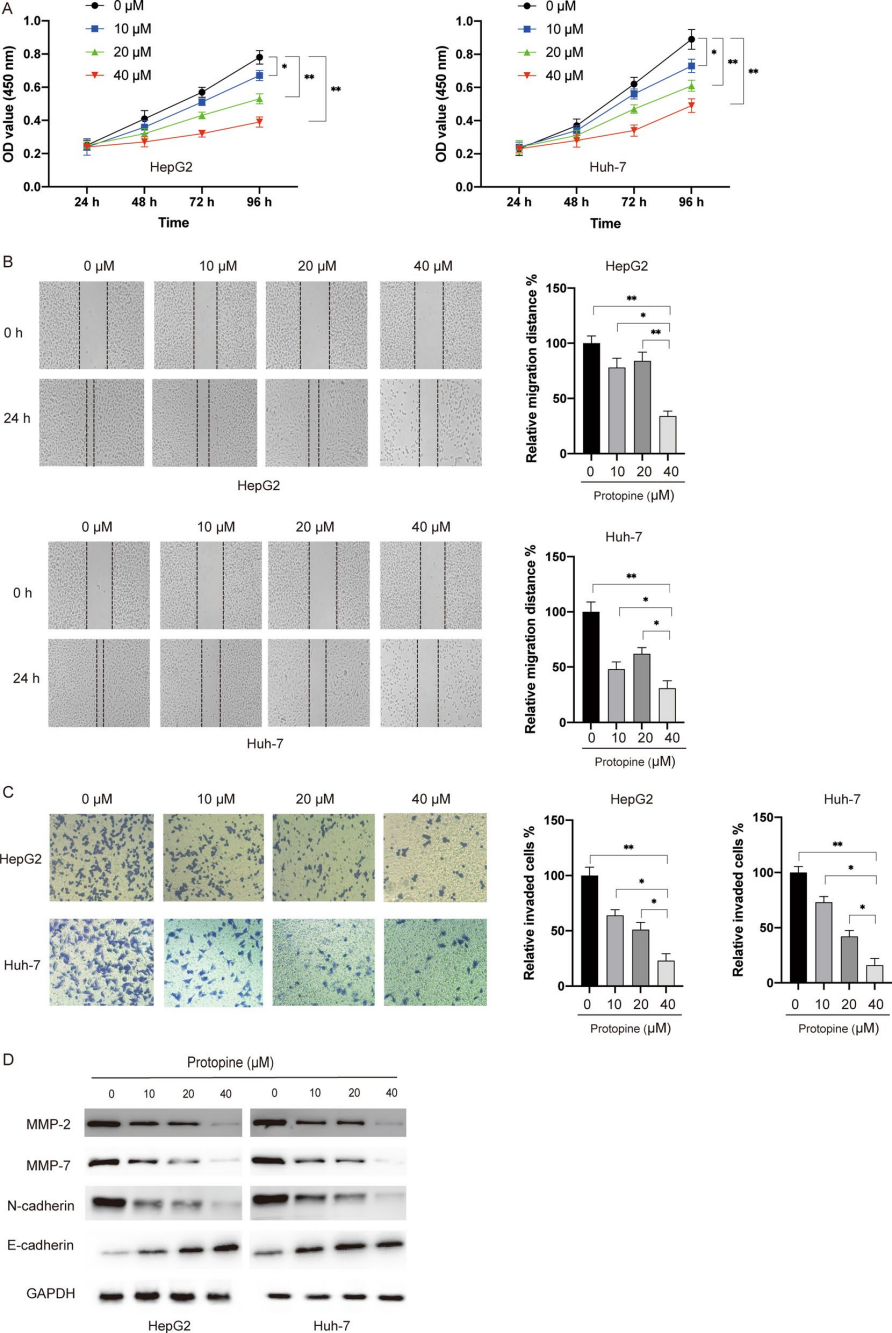
Protopine inhibited the viability, migration, invasion and EMT process of liver carcinoma cells. **A** Liver carcinoma cells HepG2, Huh7 and normal liver cell LO2 were treated with various doses of protopine (10 μM, 20 μM, 40 μM) for different time (24 h, 48 h, 72 h, 96 h), then cell viabilities were measured by the MTT assay. **B** HepG2 and Huh7 cells were treated with various doses of protopine (10 μM, 20 μM, 40 μM) for 24 h, then cellular migration was measured by the wound healing assay. **C** HepG2 and Huh7 cells were treated with various doses of protopine (10 μM, 20 μM, 40 μM) for 48 h, then cellular invasion was measured by the transwell assay. **D** HepG2 and Huh7 cells were treated with various doses of protopine (10 μM, 20 μM, 40 μM) for 24 h, then total cellular lysates were subjected to western blotting analysis with various antibodies. Data were presented as mean ± SD; *P < 0.05; **P < 0.01

Firstly, we evaluated the toxicity of protopine in two liver carcinoma cell lines (HepG2 and Huh-7). MTT assay showed that protopine inhibited the viability of both HepG2 and Huh-7 cells but not normal liver cell Lo2 in a dose- and time- dependent manner (Fig. 1A). Wound healing assay and transwell assay showed that protopine also repressed the migration and invasion of liver carcinoma cells in a dose-dependent manner (Fig. 1B and C). Given the critical roles of matrix metalloproteinases (MMPs) in the migration and metastasis of tumour cells, the levels of MMP-2 and MMP-7 were examined after exposure to protopine in liver carcinoma cells. It was found that protopine inhibited the protein levels of MMP-2 and MMP-7 in a dose-dependent manner (Fig. 1D). Since the epithelial-mesenchymal transition (EMT) process contributed to the metastasis of tumour cells, we also studied the markers of EMT. It was found that the mesenchymal marker N-cadherin was downregulated while the epithelial maker E-cadherin was upregulated after exposure to protopine in liver carcinoma cells (Fig. 1D). Taken together, those data suggested that protopine showed selectively cytotoxicity against liver carcinoma cells.

### *Protopine induced apoptosis *via* the intrinsic pathway in a caspase-dependent manner*

**Fig. 2:**
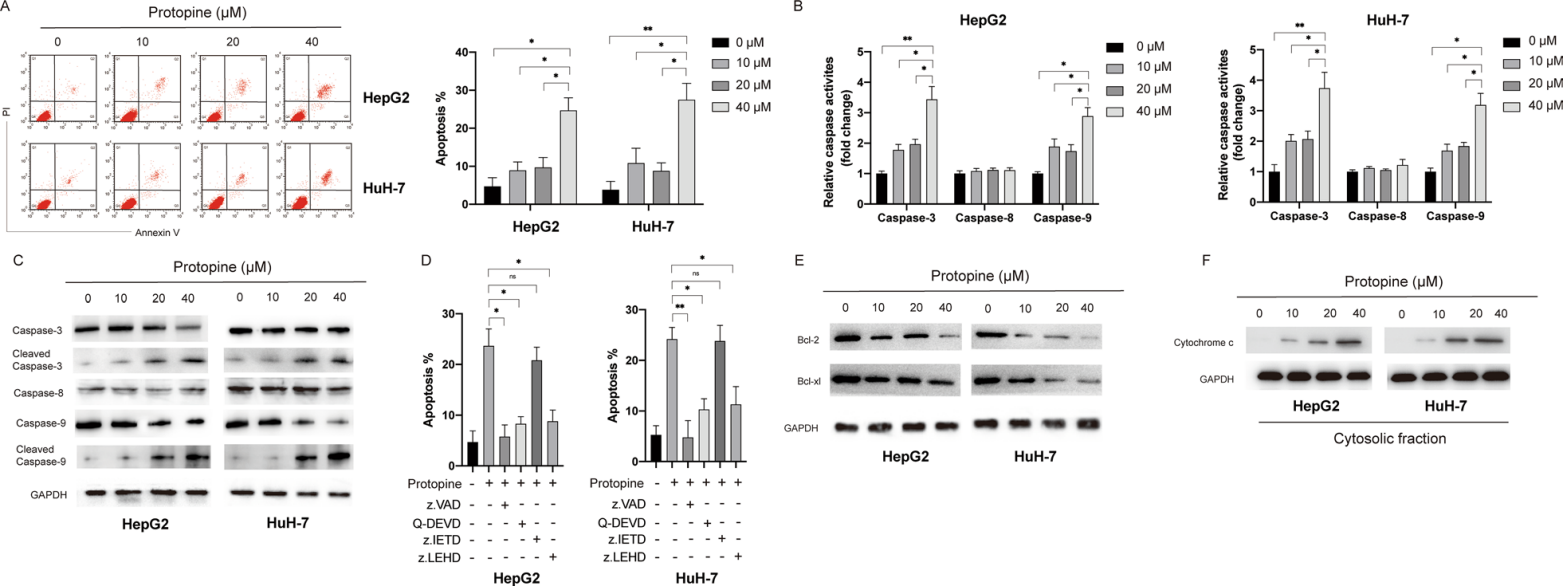
Protopine induced apoptosis via the mitochondrial pathway in a caspase-dependent manner in liver carcinoma cells. **A** HepG2 and HuH-7 cells were treated with various doses of protopine for 24 h, then cellular apoptosis was assayed by the flow cytometry. **B** HepG2 and HuH-7 cells were treated with various doses of protopine for 24 h, then relative caspases activities were measured. **C** HepG2 and HuH-7 cells were treated with various doses of protopine for 24 h, then cellular lysates were subjected to western blots with indicated antibodies. **D** HepG2 and HuH-7 cells were pretreated with different caspases inhibitors for 0.5 h, then cells were treated with protopine (40 μM) for another 24 h and cellular apoptosis was measured. **E** HepG2 and HuH-7 cells were treated with various doses of protopine for 24 h, the expression of Bcl-2 and Bcl-xl was measured by western blots. **F** HepG2 and HuH-7 cells were treated with various doses of protopine for 24 h, the release of cytochrome c into cytosol was measured by western blots. Data were presented as mean ± SD; *P < 0.05; **P < 0.01

In order to further evaluate the cytotoxicity of protopine in liver carcinoma cells, cellular apoptosis assay was performed. Annexin V-FITC assay showed that protopine induced apoptosis in a dose-dependent manner in liver carcinoma cells (Fig. 2A). In order to further confirm the apoptosis induced by protopine, relative caspases activities assay was performed. It was found that protopine treatment enhanced the activities of caspase-3 and caspase-9 but not caspase-8 in a dose-dependent manner in liver carcinoma cells (Fig. 2B). Next, the protein levels of caspases were examined by the western blots. In line with the caspases activities assays results, it was shown that protopine treatment led to the cleavage of caspase-3 and caspase-9 but not caspase-8 in a dose-dependent manner (Fig. 2C). In order to elucidate the role of caspases in the apoptosis induced by protopine, specific caspases inhibitor was applied and it was found that pan-caspase inhibitor z.VAD, specific caspase-3 inhibitor Q-DEVD and specific caspase-9 inhibitor z.LEHD but not specific caspase-8 inhibitor could block the apoptosis induced by the protopine (Fig. 2D). Considering that activation of caspase-9 and caspase-3 is a hallmark of intrinsic apoptosis which was subjected to the regulation of Bcl-2 family proteins, we also examined the expression of Bcl-2 proteins after exposure to protopine. As indicated in Fig. 2E, protopine treatment caused decrease of Bcl-2 and Bcl-xl in a dose-dependent manner in liver carcinoma cells. Furthermore, protopine treatment also triggered release of mitochondrial protein cytochrome c into the cytosol in liver carcinoma cells in a dose-dependent manner (Fig. 2F). Taken together, those data suggested that protopine induced caspase-dependent apoptosis via the mitochondrial pathway in liver carcinoma cells.

### Protopine inhibited the PI3K/Akt signalling pathway in liver carcinoma cells

**Fig. 3:**
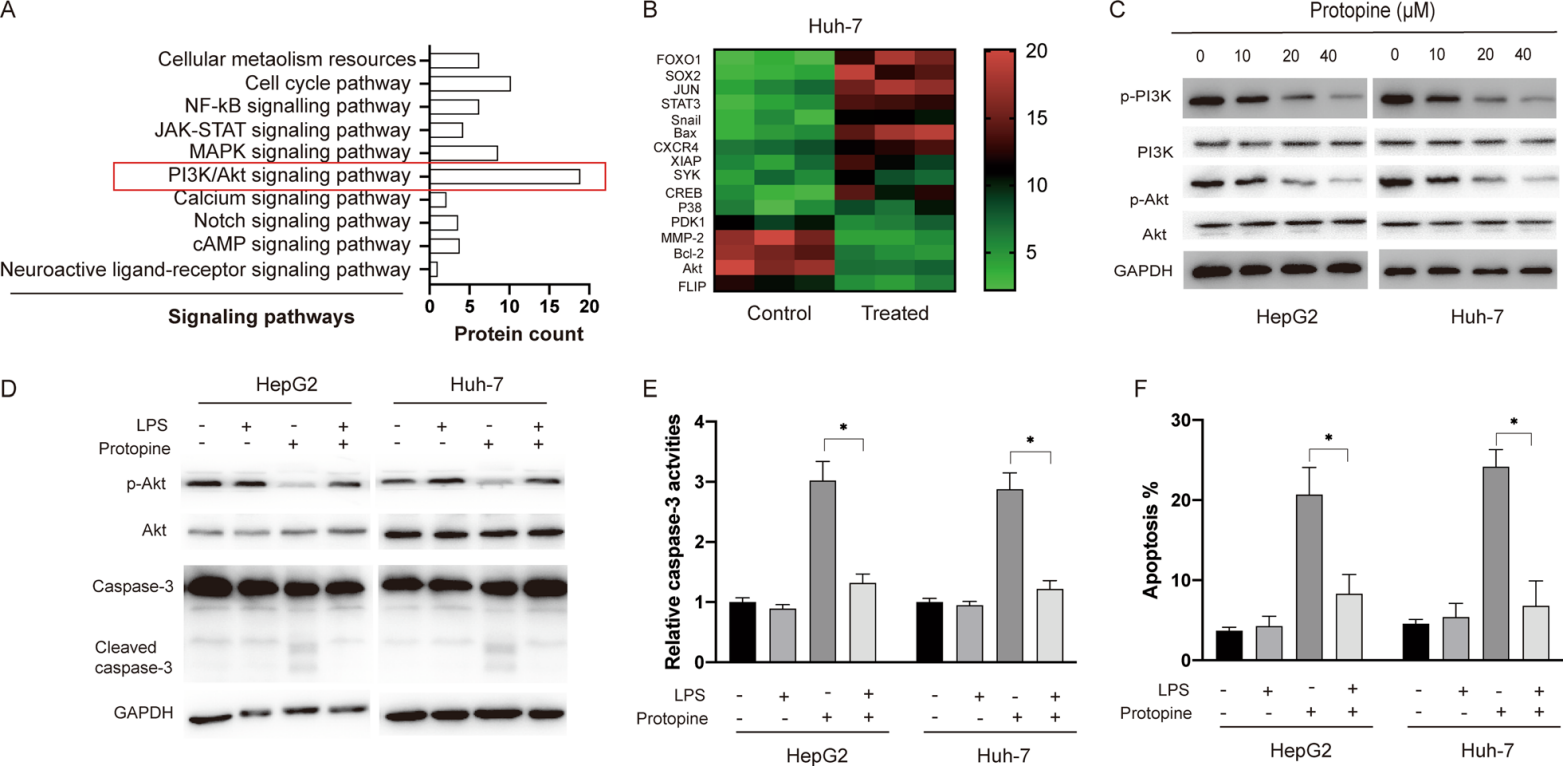
Protopine treatment inhibited the PI3K/Akt signalling pathway in liver carcinoma cells. **A** RNA-sequencing analysis showed the signalling pathways that were affected after exposure to protopine in Huh-7 cells. **B** Heatmap of the differentially expressed proteins in Huh-7 cells after treated with protopine. **C** HepG2 and Huh-7 cells were treated with various doses of protopine for 24 h, then cellular lysates were subjected to western blotting with indicated antibodies. **D** HepG2 and Huh-7 cells were pre-treated with LPS for 0.5 h then cells were treated with or without protopine for another 24 h, cellular lysates were subjected to western blotting analysis with indicated antibodies. **E** HepG2 and Huh-7 cells were pre-treated with LPS for 0.5 h then cells were treated with or without protopine for another 24 h, relative caspase-3 activities were measured. **F** HepG2 and Huh-7 cells were pre-treated with LPS for 0.5 h then cells were treated with or without protopine for another 24 h, then cellular apoptosis was measured. Data were presented as mean ± SD; *P < 0.05; **P < 0.01

In order to elucidate the detailed mechanisms underlying the apoptosis induced by protopine in liver carcinoma cells, RNA sequencing analysis was conducted. It was found that various pathways were affected after exposure to protopine in liver carcinoma and among the most enriched pathways was PI3K/Akt pathway (Fig. 3A). Furthermore, various genes that were affected in Huh7 cells after protopine treatment were presented in a heat map and it was also found that the expression of proteins related with PI3K/Akt signalling pathway was affected (Fig. 3B). Western blots results indicated that both phosphorylated PI3K and Akt were repressed after exposure to protopine in liver carcinoma cells (Fig. 3C). In order to further confirm the role of PI3K/Akt signalling pathway in the apoptosis induced by protopine, LPS.

(Lipopolysaccharide) was applied to activate the PI3K/Akt signalling pathway. As indicated in Fig. 3D, LPS treatment could reverse the inhibition of protopine on phosphorylated Akt. Meanwhile the cleavage of caspase-3 caused by protopine was abrogated by the LPS in liver carcinoma cells (Fig. 3D). Furthermore, enhanced caspase-3 activities (Fig. 3E) and apoptosis (Fig. 3F) induced by protopine could also be repressed by LPS. Taken together, those data suggested that inhibition of PI3K/Akt pathway which is essential for the apoptosis induced by protopine in liver carcinoma cells.

### Protopine treatment caused oxidative stress in liver carcinoma cells

**Fig. 4:**
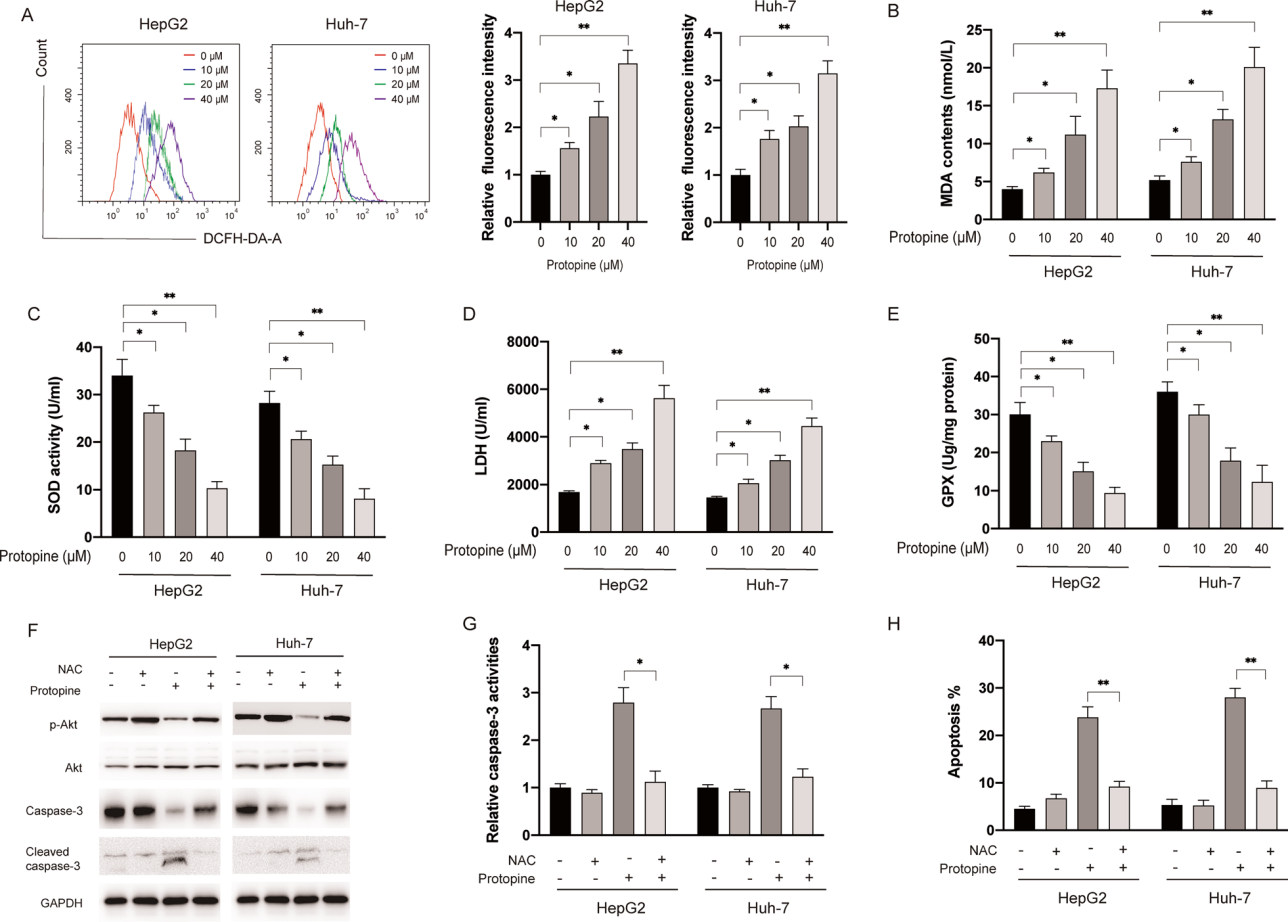
Protopine treatment led to the generation of ROS in liver carcinoma cells. **A** HepG2 and Huh-7 cells were treated with various doses of protopine for 6 h, intracellular ROS generation was measured by flow cytometer. **B** HepG2 and Huh-7 cells were treated with various doses of protopine for 6 h, then the content of MDA was measured. **C** HepG2 and Huh-7 cells were treated with various doses of protopine for 6 h, then levels of LDH were measured. **D** HepG2 and Huh-7 cells were treated with various doses of protopine for 6 h, SOD activities were measured. **E** HepG2 and Huh-7 cells were treated with various doses of protopine for 6 h, levels of GPX were measured. F, HepG2 and Huh-7 cells were pre-treated with NAC (10 mM) for 0.5 h, then cells were treated with or without protopine for 24 h, cellular lysates were subjected to western blotting analysis with indicated antibodies. **G** HepG2 and Huh-7 cells were pre-treated with NAC for 0.5 h then cells were treated with or without protopine for another 24 h, relative caspase-3 activities were measured. **H** HepG2 and Huh-7 cells were pre-treated with NAC for 0.5 h then cells were treated with or without protopine for another 24 h, then cellular apoptosis was measured. Data were presented as mean ± SD; *P < 0.05; **P < 0.01

It was well documented that various alkaloids could triggered oxidative stress which is essential for the cytotoxicity against tumour cells [12]. In order to examine whether protopine affected the oxidative stress in liver carcinoma cells, the fluorescent probe DCFH-DA was applied to study the effect of protopine on the generation of ROS in liver carcinoma cells. As shown in Fig. 4A, protopine treatment significantly increased cellular ROS levels in both HepG2 and Huh-7 cells. Furthermore, it was also found protopine treatment led to the increase of MDA and LDH while SOD and GPX activities were inhibited by protopine in liver carcinoma cells (Fig. 4B–E). In order to further elucidate the role of ROS in the apoptosis triggered by protopine, a well-known ROS scavenger NAC was applied. As shown in Fig. 4F, NAC treatment reversed the effects of protopine on the phosphorylation of Akt and caspase-3 cleavage in liver carcinoma cells. In addition, increased caspase-3 activities (Fig. 4G) and apoptosis (Fig. 4H) induced by protopine also be abrogated by NAC in liver carcinoma cells. Taken together, those data suggested that generation of ROS is essential for the apoptosis and inhibition of PI3K/Akt induced by protopine in liver carcinoma cells.

### *Protopine inhibited liver carcinoma cells growth *in vivo

**Fig. 5:**
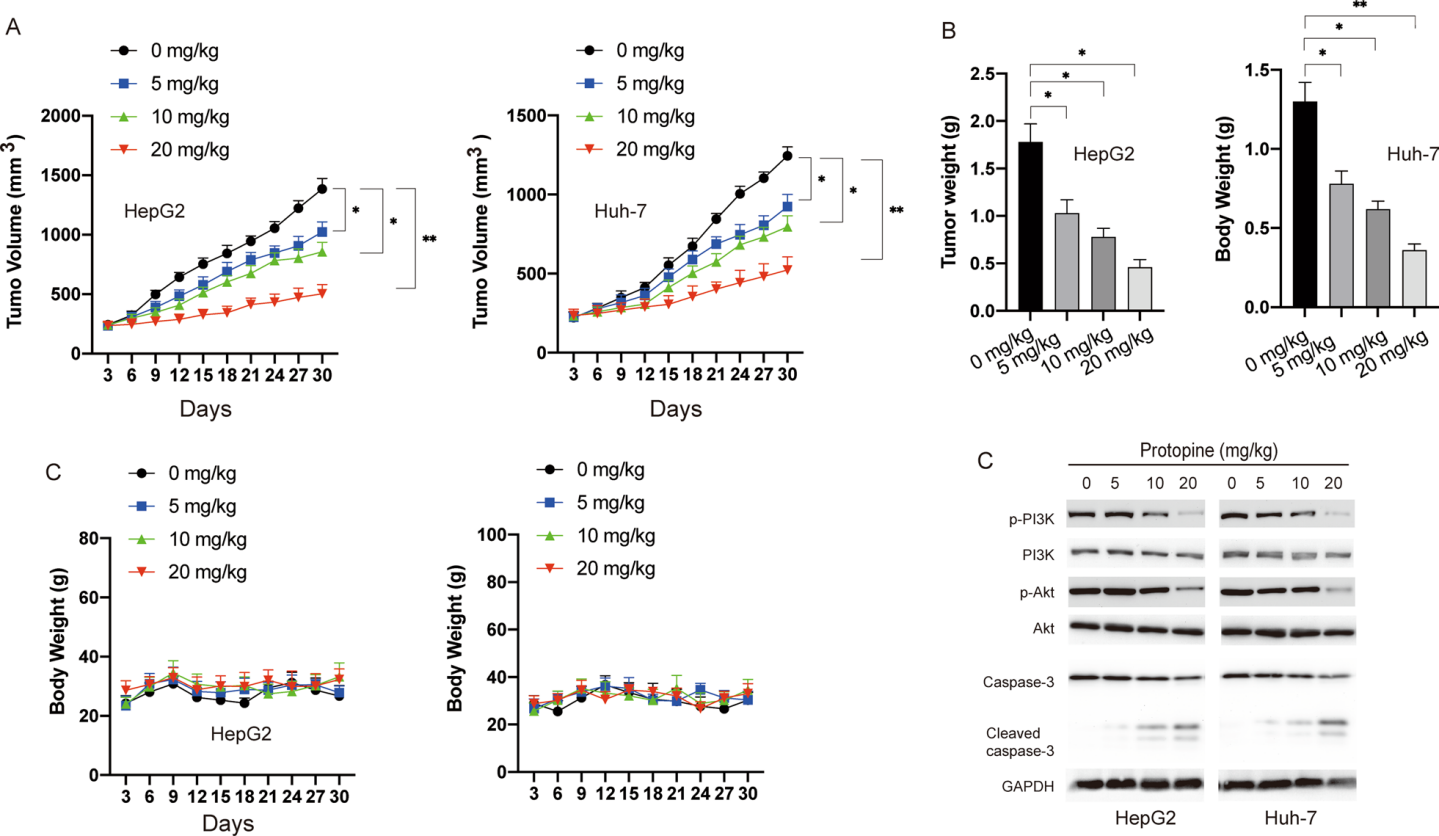
The effect of protopine on the tumour growth in xenograft mice. **A** tumour volume change in HepG2 and Huh-7 group after treated with protopine. **B** tumor weight was measured after mice were sacrificed. **C** body weights of mice were measured during the study. **D** tumours were resected and subjected to western blotting with indicated antibodies. Data were presented as mean ± SD; *P < 0.05; **P < 0.01

We further assessed the anti-tumour effects of protopine in vivo by using the xenograft tumour model in BALB/c nude mice. Xenograft tumours were created by subcutaneous injection of HepG2 or Huh-7 cells in BALB/c mice. After solid tumour size reached over 100 mm^3^, mice were randomly divided into four groups and treated with saline or various doses of protopine (5 mg/kg, 10 mg/kg, 20 mg/kg). As shown in Fig. 5A, administration of protopine significantly inhibited the growth of liver carcinoma cells in vivo. 30 days later, mice were sacrificed and the tumours weight were measured. Tumour weight was significantly reduced in the groups treated with protopine compared with the control group (Fig. 5B). In addition, there was no obvious loss of body weight in the protopine treated groups (Fig. 5C). Finally, the xenograft tumour tissues were subjected to western blotting analysis. Similar to the in vitro results, treatment of protopine also led to the inhibition of PI3K/Akt and cleavage of caspase-3 in vivo. Taken together, those data suggested that protopine inhibited the growth of liver carcinoma cells growth in vivo.

## Discussion

Liver carcinoma is a common tumour with high incidence and mortality. Due to lack of effective therapeutic options, its urgent to identify novel potential anti-tumour agents to treat liver carcinoma. In the present study, we showed that protopine could inhibit viability and induce apoptosis of liver carcinoma cells in a caspase-dependent manner. In addition, it was also found that inhibition of PI3K/Akt signalling pathway which relied on the generation of ROS was involved in the apoptosis-induced by protopine. To our knowledge, this is the first study to indicate that protopine inhibits the viability and induces apoptosis of liver carcinoma cells both in vitro and in vivo.

Recently, protopine, an isoquinoline alkaloid, has been shown to exert cytotoxic effects against human cancer cells. For instance, protopine was able to induce both apoptosis and autophagy of colon cancer cells through stabilizing p53 [7]. In our scenario, we also found that protopine showed strong cytotoxicity against liver carcinoma cells but not normal liver cells. Liver carcinoma are often diagnosed too late to be operable. During the metastasis, malignant liver carcinoma cells usually gain migratory and invasive ability and loss junctions between cells [13]. This phenotypic change of epithelial tumour cells was known as the epithelial-to-mesenchymal transition (EMT) process. Multiple proteins such as MMP-2/7 play essential roles in the migration and invasion of tumour cells through regulation of degradation of extracellular matrix proteins [14]. In this study, it was found that protopine treatment inhibited the migration and invasion of liver carcinoma cells. Furthermore, it was also observed that protopine treatment inhibited the MMP-2/7 and EMT biomarkers. Those findings suggested that protopine might possess the inhibitory effect on the metastasis of liver carcinoma cells and it would be interesting to test it in more types of carcinoma cells.

Apoptosis is also known as the type I programmed cell death which is characterized by multiple biophysical changes such as DNA fragmentation and nuclear condensation [4, 11]. Apoptosis can be triggered via two pathways namely the extrinsic and the mitochondrial pathway. The extrinsic and mitochondrial apoptotic pathway can be initiated by the caspase-8 and caspase-9, respectively. Both the activation of caspase-8 and caspase-9 will lead to the activation of caspase-3 which acts as an executioner of apoptosis [15]. Our results showed that protopine treatment led to the activation of caspase-9 and caspase-3 but not caspase-8. In addition, the release of mitochondrial protein cytochrome *c* into cytosol was also observed after exposure to protopine. Those data indicated that protopine triggered apoptosis via the mitochondrial pathway in liver carcinoma cells. Our findings are in line with previous studies which showed that protopine also induced apoptosis via the mitochondrial apoptotic pathway in the prostate and colon cancer cells [7, 8].

The essential role of PI3K/Akt signalling pathway has been implied in liver carcinoma development and progression both in vitro and in vivo [16]. PI3K/Akt signalling pathway is closely correlated with various biological activities such as cell proliferation, differentiation, metastasis, tumorigenesis and cell death [17]. By RNA-seq analysis, the PI3K/Akt signalling pathway was identified to be markedly altered in Huh-7 cells after exposure to protopine. Hence, PI3K/Akt signalling pathway was chosen for further investigation. By using LPS, an activator of PI3K/Akt, we found that inhibition of PI3K/Akt is critical for the apoptosis induced by protopine in liver carcinoma cells. To our knowledge, it’s the first time indicated that protopine could act as an inhibitor of PI3K/Akt signalling pathway. Considering that targeting PI3K/Akt signalling pathway is a promising strategy for the treatment of various cancers, it would be meaningful to examine the inhibitory effect of protopine on PI3K/Akt in more types of cancer cells.

Amounting evidence indicates that plenty of natural products exert anti-tumour effects via regulation of the oxidative stress in tumour cells [18]. We found that protopine treatment not only increases the levels of ROS, MDA and LDH but also inhibits the SOD activity and GPX levels. Meanwhile, ROS scavenger could abrogate the apoptosis induced by protopine. It was well-documented that multiple natural products induced apoptosis via accumulation of ROS which act as an inhibitor of PI3K/Akt signalling pathway [19]. Similar to previous studies, we found that ROS generation induced by protopine is critical for the inhibition of PI3K/Akt signalling pathway. Recently, some studies showed that prolonged chemotherapy decreases the intracellular levels of ROS which may led to chemoresistance in the tumours cells [20]. Therefore, application of agents that able to repress antioxidant system and to enhance intracellular ROS levels might be a promising strategy to trigger cell death and/or overcome chemoresistance of tumours cells [21]. Based on that, it would be interesting to test if protopine could be used as a useful adjuvant in the treatment of cancers. Taken together, our data suggest that the generation of ROS by protopine plays a critical role in the apoptosis and acts as an upstream regulator of PI3K/Akt signalling pathway in liver carcinoma cells.

We also showed that administration of protopine inhibited the tumour growth in xenograft mice. Similar to the in vitro results, inhibition of PI3K/Akt and activation of caspase-3 were also observed in vivo. In addition, there were little significant difference of body weights between the control group and protopine treated groups, indicating that protopine can be well tolerant. However, there’s some limitations of our study. Firstly, RNA-sequencing results showed that protopine affects various signalling pathways in liver carcinoma cells, it would be interesting to explore the roles of other signalling pathways in the biological activities of protopine. Secondly, whether protopine could repress the metastasis was not investigated in this study and it would be meaningful to study in the future.

## Conclusions

In summary, we have examined the potential anti-tumour effects of protopine on two human liver carcinoma cell lines both in vitro and in vivo. Protopine inhibits tumour cells viability, induces caspase-dependent apoptosis via the intrinsic pathway and induces ROS which further blocks PI3K/Akt signalling pathway. Noteworthy, a very recent study showed that protopine has hepatoprotective activity in vivo [22]. Therefore, protopine may be developed as an effective agent to treat liver carcinoma by further investigation.

## Data Availability

Not applicable.
